# IL-4 induces reparative phenotype of RPE cells and protects against retinal neurodegeneration via Nrf2 activation

**DOI:** 10.1038/s41419-022-05433-0

**Published:** 2022-12-20

**Authors:** Tian Zhou, Ziqi Yang, Biyan Ni, Hong Zhou, Huiyi Xu, Xiaojing Lin, Yingmin Li, Chunqiao Liu, Rong Ju, Jian Ge, Chang He, Xialin Liu

**Affiliations:** State Key Laboratory of Ophthalmology, Zhongshan Ophthalmic Center, Sun Yat-sen University, Guangdong Provincial Key Laboratory of Ophthalmology and Visual Science, 510060 Guangzhou, China

**Keywords:** Interleukins, Neurodegeneration

## Abstract

Retinal degeneration is a kind of neurodegeneration characterized by progressive neuronal death and dysfunction of retinal pigment epithelium (RPE) cells, leading to permanent visual impairment. It still lacks effective therapeutic options and new drugs are highly warranted. In this study, we found the expression of IL-4, a critical regulator of immunity, was reduced in both patients and mouse models. Importantly, exogenous intravitreal IL-4 application could exert a novel neuroprotective effect, characterized by well-preserved RPE layer and neuroretinal structure, as well as amplified wave-amplitudes in ERG. The RNA-seq analysis revealed that IL-4 treatment suppressed the essential oxidative and pro-inflammatory pathways in the degenerative retina. Particularly, IL-4 upregulated the IL-4Rα on RPE cells and induced a reparative phenotype via the activation of Nrf2 both in vitro and in vivo. Furthermore, the Nrf2-/- mice displayed no recovery in response to IL-4 application, highlighting a significant role of Nrf2 in IL-4-mediated protection. Our data provides evidence that IL-4 protects against retinal neurodegeneration by its antioxidant and anti-inflammatory property through IL-4Rα upregulation and Nrf2 activation in RPE cells. The IL-4/IL-4Rα-Nrf2 axis maybe the potential targets for the development of novel therapies for neurodegenerative diseases.

## Introduction

Retinal degenerative diseases are a heterogeneous group of progressive neurologic disorders, including age-related macular degeneration (AMD) and retinitis pigmentosa (RP), which are worldwide leading causes of irreversible blindness. These diseases are characterized by progressive degeneration of the retinal pigment epithelium (RPE) and/or overlying photoreceptors [1, 2]. RPE is injured in response to oxidative stress and there is lack of effective anti-oxidative agents for RPE protection [3]. In clinical trials, the effect of anti-oxidative diet therapy is limited in most cases, and the trials on replacing dysfunctional/damaged RPE are still in progress [4–6].

IL-4, a pleiotropic cytokine, plays an essential role in the regulation of a variety of immune responses, including Th2 cell-mediated immunity, IgE class switching in B cells, and macrophage activation towards M2 phenotype [7–10]. Targeting IL-4 in Th2 cell-mediated immune response has led to the clinical application of IL-4 antagonist, dupilumab, in treatment of atopic dermatitis [11]. Meanwhile, the action of IL-4-induced M2 macrophage transition makes IL-4 a potential therapeutic target for macrophage-mediated diseases [12–14]. For instance, IL-4 improves myocardial repair and enhances function of damaged hearts through induction of M2-reparative macrophages [15]; Clinical trials targeting IL-4 has also been demonstrated safe for cancer treatment [16]. The IL-4 signaling is mediated through the IL-4R α-chain (IL-4Rα), which dimerizes either with the common γ-chain or with the IL-13Rα, constituting type I or II IL-4 receptors, respectively [17]. The IL-4Rα expression is not limited to the hematopoietic T cells, B cells and macrophages, but also in endothelial cells, fibroblasts, and RPE cells [18–20]. It would be valuable to know whether IL-4/IL-4Rα signaling is also involved in functions and related diseases of these cell types, thus to extend the repertoire of IL-4 clinical applications.

In this study, we investigated the role of IL-4 on RPE cells in two retinal neurodegeneration mouse models, including the sodium iodate (NaIO_3_)-induced mouse and the inherited rd10 mouse [21]. We observed excessive inflammation responses in the NaIO_3_-induced retinae with downregulation of IL-4 expression. The choroid complex of AMD patients and rd10 mice also displayed decreased IL-4 expression. Supplementing IL-4 was able to rescue RPE cells and protect against the degenerative retina in both NaIO_3_ induced retinae and rd10 mice. The salient protective property of IL-4 points to a novel therapeutic approach for treating retinal degenerative diseases. We further found the IL-4/IL-4Rα-Nrf2 axis being crucial for protecting RPE cells against inflammation and oxidative stress.

## Results

### Aberrant inflammation and reduction of IL-4 expression in the degenerative retinae

**Fig. 1:**
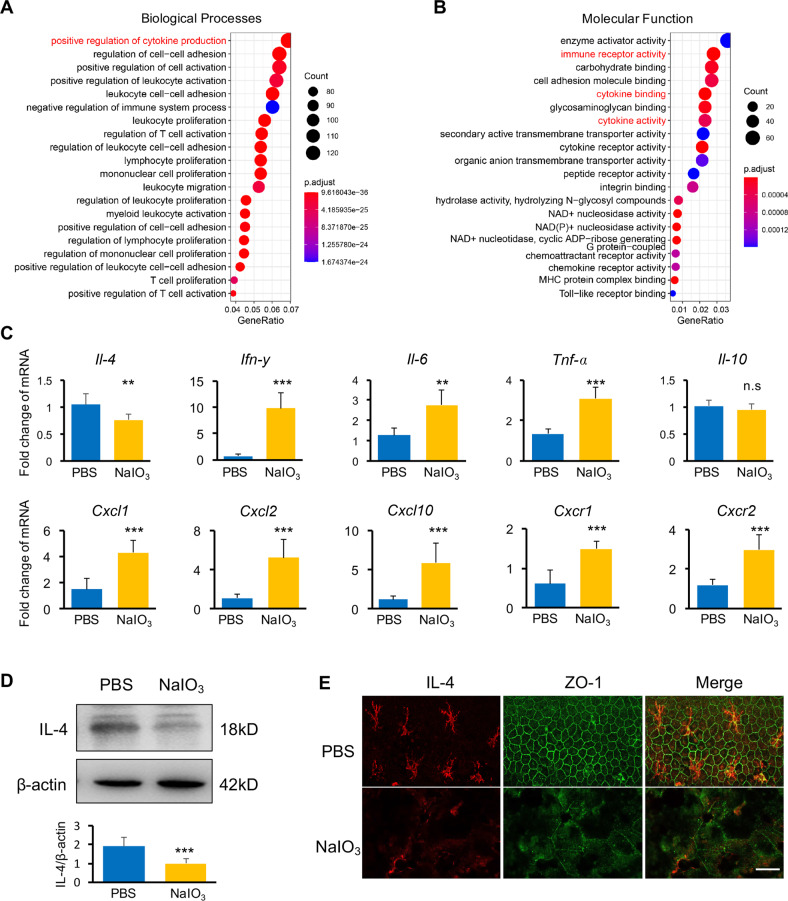
NaIO_3_-induced degenerative retinae underwent inflammatory responses and displayed decreased IL-4 expression. **A**, **B** GO enrichment of the upregulated DEGseq in NaIO_3_-induced degenerative retinae showed the enriched differentially expressed mRNAs in biological processes (**A**) and molecular function (**B**). **C** qPCR analysis showed decreased mRNA levels of IL-4 in NaIO_3_-induced degenerative retina, while the pro-inflammatory cytokines and chemokines/chemokine receptors, including *Ifn-γ*, *Il-6*, *Tnf-α*, *Cxcl1*, *Cxcl2*, *Cxcl10*, *Cxcr1* and *Cxcr2* were significantly increased. The anti-inflammatory *Il-10* had no significant difference between the NaIO_3_-induced retina and normal control, *n* = 3. **D** Western blotting analysis showed marked decreased IL-4 expression in the NaIO_3_-retinae in comparison to controls, *n* = 3. **E** Lack of IL-4 expression (red) and broken ZO-1-positive RPE barrier (green) were observed in the NaIO_3_-induced RPE flat-mount sheet by immunohistochemistry analysis. Scale bar, 50 µm. *n* = 3. Data are shown as mean ± SEM, **P < 0.01, ***P < 0.001, n.s: not significant, unpaired Student’s *t*-test in **C** and **D**.

Inflammation is a hallmark of retinal degenerative diseases [22]. To obtain the gene expression landscape of inflammation in retinal degeneration, thereby identify potential mediators, we aimed to take an unbiased experimental approach of deep sequencing of the RPE/choroid/sclera complex, the main pathological lesions in the NaIO_3_-induced retinal degeneration model. NaIO_3_, a stable oxidizing agent, can trigger the accumulation of reactive oxygen species (ROS) in RPE cells by converting glycine to potentially toxic glyoxylate, resulting in rapid ablation of RPE and consequent photoreceptor loss, resembling the degeneration progression in the dry AMD [23, 24]. The Gene Ontology analysis of the entire set of upregulated genes in NaIO_3_ group revealed the significant enrichment for terms related to regulation of cytokine production (Fig. 1A), immune receptor activity, and cytokine activity (Fig. 1B) among the top ones.

Next, we performed the qPCR to validate the expressions of common cytokines. Interestingly, among the eight cytokines or chemokines tested, IL-4 was the only one significantly reduced in the NaIO_3_-induced RPE/choroid/sclera complex while the others were either drastically elevated, such as *Ifn-γ*, *Il-6*, *Tnf-α*, *Cxcl1*, *Cxcl2*, and *Cxcl10*, or unchanged (*Il-10*) (Fig. 1C). The expressions of chemokines receptors *Cxcr1* and *Cxcr2* were also increased (Fig. 1C). The lower expression of IL-4 in the NaIO_3_-treated group of the RPE/choroid/sclera was further confirmed at protein level by Western blotting (Fig. 1D). Immunohistochemistry analysis of flat-mount RPE sheet further validated very low level of IL-4 expression in the NaIO_3_-induced RPE monolayer (Fig. 1E).

In addition, we analyzed a published single-cell transcriptomics data of human RPE and choroid from the health and macular degeneration patients [25, 26] (GSE135922 from NCBI GEO database). The cells were clustered into RPE, endothelial cells, pericytes, fibroblasts, melanocytes, schwann cells, and immune cells (Fig. S1A, B). The violin plot data showed that IL-4 was mainly enriched in immune cells and was particular decreased in the sample from macular degeneration patients (Fig. S1C, D). We further interrogated those immune cells into different types, including microglia/macrophage, NK/T cells, B cells, mast cells, and others with respective makers according to previous studies [25, 27, 28]. Interestingly, IL-4 was mainly expressed in microglia/macrophage (Fig. S1E), which are consistent with the branchy morphology of the microglia in Fig. 1E. The RPE-sclera complex from rd10 mice also displayed decreased IL-4 expression in comparison to the same-age wild type mice (Fig. S3A). These data collectively suggested that the reduction of IL-4 expression involved in the process of retinal degeneration.

### Exogenous IL-4 preserved neuroretinal structure and function in the degenerative retina

**Fig. 2:**
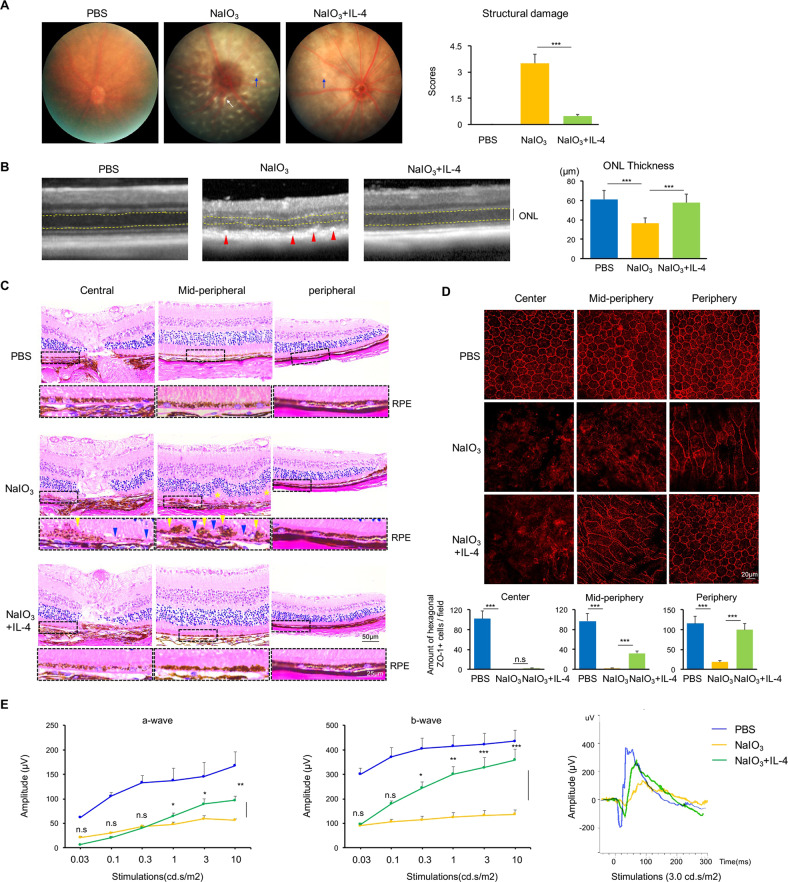
IL-4 protected neuroretinal structure and function in the NaIO3-induced retinal degenerative mice. **A** The funduscopy of NaIO_3_-induced degenerative retina was characterized by pale appearance and some dot-like or linear lesions showing structural damage (blue arrows), which were alleviated after IL-4 treatment. *n* = 6. **B** Representative images of the OCT scanning. The NaIO_3_ model demonstrated obvious discontinuity of RPE layer with high-reflective deposits (red arrows), while IL-4-treated RPE exhibited as a smooth, continuous, and uniform reflectiveness layer. The ONL thickness (distance between yellow lines) was calculated and compared. *n* = 6. **C** H&E staining showed crumby-structure patches and melanin rich aggregation in the RPE layer of the NaIO_3_-retina, particularly in the mid-peripheral locations. After IL-4 treatment, the RPE layer was preserved and well-organized with less melanin rich aggregation and the retinal folds were relieved. Scale bar: 50 μm and 25 μm. **D** Representative images of RPE flat-mount in the center, mid-periphery and periphery areas sheet showing RPE integrity by ZO-1 staining. NaIO_3_ disrupted the well-organized hexagonal-shaped RPE cells, whereas IL-4 treatment could preserve it with more organized barrier structures specifically in the mid-periphery and periphery areas. Statistical analysis showed the amount of ZO-1+ cells. *n* = 6, Scale bar: 20 μm. **E** ERG examinations showed a- and b-waves were almost extinct in NaIO_3_-retina. However, IL-4 treatment protected the neuroretinal functions with increased amplitudes of both a- and b-waves. *n* = 6. Data are shown as mean ± SEM, ***P < 0.001, n.s: not significant, one-way ANOVA test.

Given IL-4 as the critical decreased cytokine in the degenerative retina in addition to its anti-inflammation function, we wondered whether IL-4 supplement might be protective for the retinal degeneration. To test this speculation, IL-4 was injected intravitreally to the NaIO_3_-induced mice and rd10 mice. As shown in Fig. 2A, pale-colored retinae with structural damages such as white dot-like or linear lesions were observed by funduscopy in NaIO_3_ injected retinae contrasting to that of PBS-injected reddish-orange controls; whilst IL-4 treatment significantly alleviated these pathological changes. When performed OCT scan, RPE band in the NaIO_3_-treated eye exhibited aberrant hyperreflective discrete foci (Fig. 2B, red arrows), indicating the RPE lesions. In contrast, the IL-4-treated RPE exhibited as a smooth, continuous, and uniform reflectiveness RPE band, more close to that of the PBS-treated controls (Fig. 2B). In addition, the reduced thickness of ONL in NaIO_3_-induced retina was recovered after IL-4 treatment (Fig. 2B). Consistently, many crumby-structure patches (yellow arrows), possibly the pathologic aggregation of melanin in the RPE cells, were observed in the NaIO_3_-retina in H&E staining (Fig. 2C). Besides, the discontinue degenerated RPE layer was usually associated with retinal folds (yellow stars) in the NaIO_3_-retina, which were severer in the mid-periphery location than those in the center or periphery (Fig. 2C). Notably, IL-4 treatment largely preserved the well-organized RPE layer and inhibited the associated photoreceptor degeneration, with less melanin rich aggregation and ONL folds (Fig. 2C). On flat-mount RPE sheet, NaIO_3_ disrupted the hexagonal-packaging of RPE cells and barrier as indicated by a tight junction marker, ZO-1 staining, particular in the center and mid-periphery. IL-4 treatment again remarkably rescued these pathogenic phenotypes (Fig. 2D). Finally, ERG analysis was used to determine neuroretinal function after IL-4 treatment. Remarkably, the typically reduced amplitudes of a-wave and b-wave in NaIO_3_-induced retina were restored by IL-4 treatment (Fig. 2E). A spontaneous retinal degeneration rd10 mouse model was also treated by IL-4 intravitreal injection. Similarly, IL-4 treatment improved retinal neurofunction of rd10 mice with increased amplitudes in ERG (Figure S2A) and restored vision in optomotor task (Fig. S2B). In H&E staining, the IL-4 treatment group displayed increased ONL thickness (Fig. S2C). Taken together, the IL-4 treatment exhibits a neuroprotective effect in the retinal degenerative process.

### Transcriptome analysis revealed the inhibiting effects of IL-4 on inflammatory and oxidative stress pathways

**Fig. 3:**
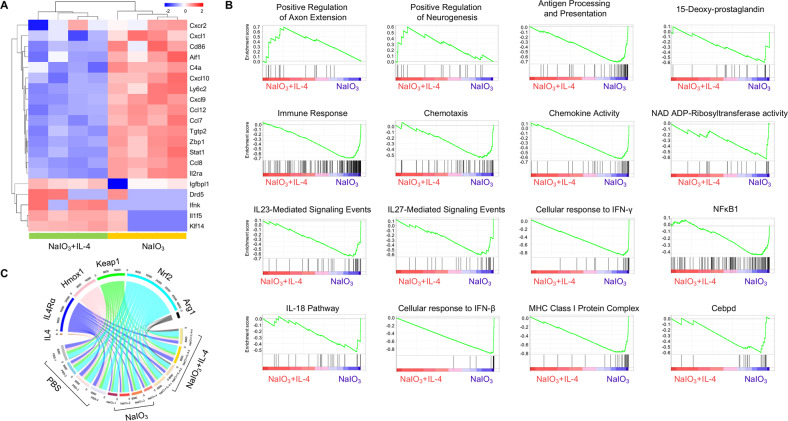
The transcriptional changes of NaIO_3_-induced RPE sheet. **A** Heatmap showing differentially expressed inflammation-related genes in NaIO_3_ mice with and without IL-4 treatment. The pro-inflammatory genes such as *Cxcr2*, *Cxcl1*, *Cxcl10*, *Stat1*, *Ccl8*, etc were down-regulated in the IL-4-treated group, while the anti-inflammatory genes, including *Il1f5*, *Klf14*, *Drd5*, *Ifnk*, etc were upregulated. **B** GSEA analysis revealed significantly enriched gene sets such as axon extension and neurogenesis in the IL-4 group, and the enriched gene sets, including the inflammation and oxidative stress-related gene sets in the NaIO_3_-RPE without IL-4. *n* = 3, FDR value <0.25. **C** The enrichment analysis of IL-4Rα and anti-oxidative genes such as *Nrf2*, *Hmox1*, and *Arg1* in different groups were presented by circos plot. The thickness and color of the ribbons correlated to the genes expression.

Next, we examined the transcription profile of IL-4-treated degenerative retina in order to find out the underlying mechanism. The RNA was extracted from the NaIO_3_-induced RPE/choroid/sclera complex with or without IL-4 treatment followed by RNA-seq assay. In total, 7663 differentially expressed genes were identified when the NaIO_3_ + IL-4 treatment and NaIO_3_ control group were compared. Among these genes, 3567 (46.5%) genes were upregulated whereas 4096 (53.45%) were down-regulated upon the IL-4 treatment. In particular, a list of pro-inflammatory genes such as *Cxcr2*, *Cxcl1*, *Cxcl10*, *Stat1*, *Ccl8* were down-regulated, while the anti-inflammatory genes, including *Il1f5*, *Klf14*, *Drd5*, *Ifnk* were upregulated after IL-4 treatment (Fig. 3A). In addition, the GSEA analysis revealed that the genes involved in positive regulation of axon extension and neurogenesis including Lrp1, Ntrk3, Ntrk2, Ntn1, Wnt2, Serpine2, and Notch1 were enriched in the IL-4-treated degenerative retina (Fig. 3B), indicating the possible neuroprotective effects of IL-4. Particularly, in comparison to NaIO_3_ control group, IL-4 treatment suppressed the expressions of pro-inflammatory or pro-oxidative stress enriched gene sets, including antigen processing and presentation, immune response, chemotaxis, chemokine activity, IL-23-mediated signaling, cellular response to IFN-γ or IFN-β, NAD ADP-Ribosyltransferase activity and so on (Fig. 3B). These results further suggested that IL-4 supplements could suppress the inflammation or oxidative stress in the degenerative retina and orchestrate an immune niche for neuroprotection.

### IL-4 drove the expression of IL-4Rα and Nrf2

**Fig. 4:**
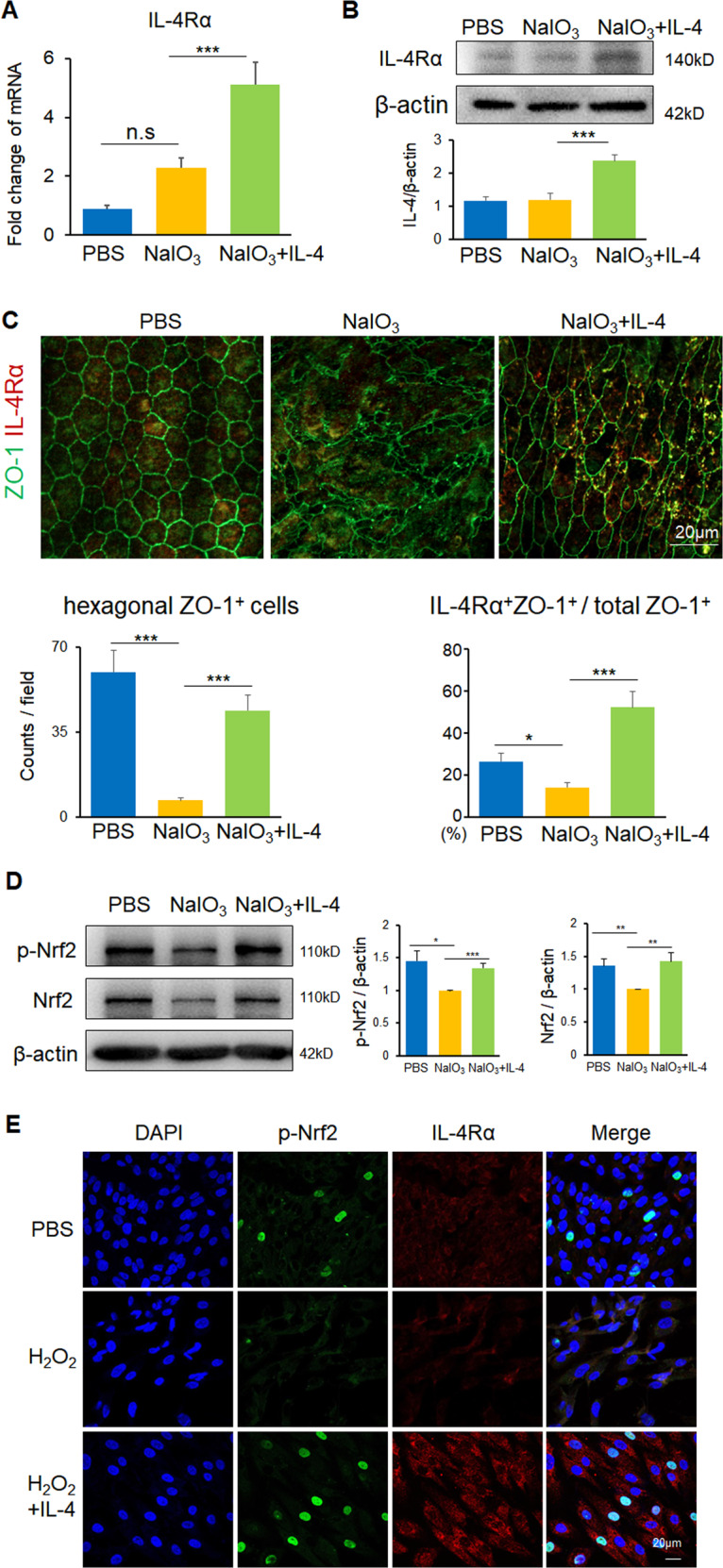
IL-4R and Nrf2 activation in response to IL-4 in vivo and in vitro. **A**, **B** qPCR (**A**) and western blotting analysis (**B**) showed elevated IL-4Ra expression in RPE complex from the NaIO_3_-induced degenerative mice after IL-4 treatment. *n* = 3. **C** Representative images of RPE flat-mount sheet revealed that increased IL-4Ra distributed alongside the RPE tight junction marker ZO-1. Scale bar: 20 μm. The hexagonal ZO-1+ cells per field and the percentage of IL-4Rα + ZO-1+ cells and total ZO-1+ cells were calculated for statistics. **D** Western blotting analysis showed reduction of total and phosphorylated Nrf2 expression in NaIO_3_ model, while IL-4 treatment induced their elevations. *n* = 3. **E** Representative images of cultured RPE cells under oxidative stress insults showing lack of IL-4Rα and phosphorylated Nrf2 expression, which were induced after IL-4 stimulation. Data are shown as mean ± SEM, *P < 0.05, **P < 0.01, ***P < 0.001, n.s: no significance, one-way ANOVA test.

Further, the circos plot analysis of the RNA-seq data revealed a positive correlation between the expression of IL-4Rα and antioxidant genes such as *Nrf2*, *Hmox1*, and *Arg1* (Fig. 3C). IL-4 usually binds to the high-affinity subunit of IL-4Rα to induce immune response [19]. Here we found the IL-4Rα was highly expressed in the IL-4-treated RPE complex both in mRNA and protein level (Fig. 4A, B and Fig. S3A). IL-4-flat-mount RPE sheet revealed that the IL-4Rα distributed alongside the ZO-1, indicating the possible involvement of IL-4/IL-4Rα axis in retinal neuroprotection (Fig. 4C and Fig. S3B). In addition, oxidative stress contributes to a range of neurodegenerative diseases [29–31]. Nrf2, a transcription factor, which is essential for protection against oxidative stresses, has been known to attenuate degeneration and inflammation [32]. Here we found that whereas the total and phosphorylated expression levels of Nrf2 were reduced in the NaIO_3_-induced RPE/choroid/sclera complex when compared to the ones in the PBS control, the addition of IL-4 increased their levels closed to those in the control (Fig. 4D). In the rd10 mice, phosphorylated Nrf2 was decreased in the PBS control group, whereas increased Nrf2 phosphorylation level was observed after IL-4 treatment (Fig. S3A, B). Consistently, in the in vitro experiments, H_2_O_2_, a usually used oxidative stress inducer, decreased the levels of p-Nrf2 and IL-4Rα in the cultured RPE cells, which were restored after IL-4 treatment (Fig. 4E). These results indicate that Nrf2 is probably implicated in the reparative and protective role of IL-4.

### Nrf2 deficiency abrogated the neuroprotective effect of IL-4 in NaIO_3_-induced retinal degeneration mice

**Fig. 5:**
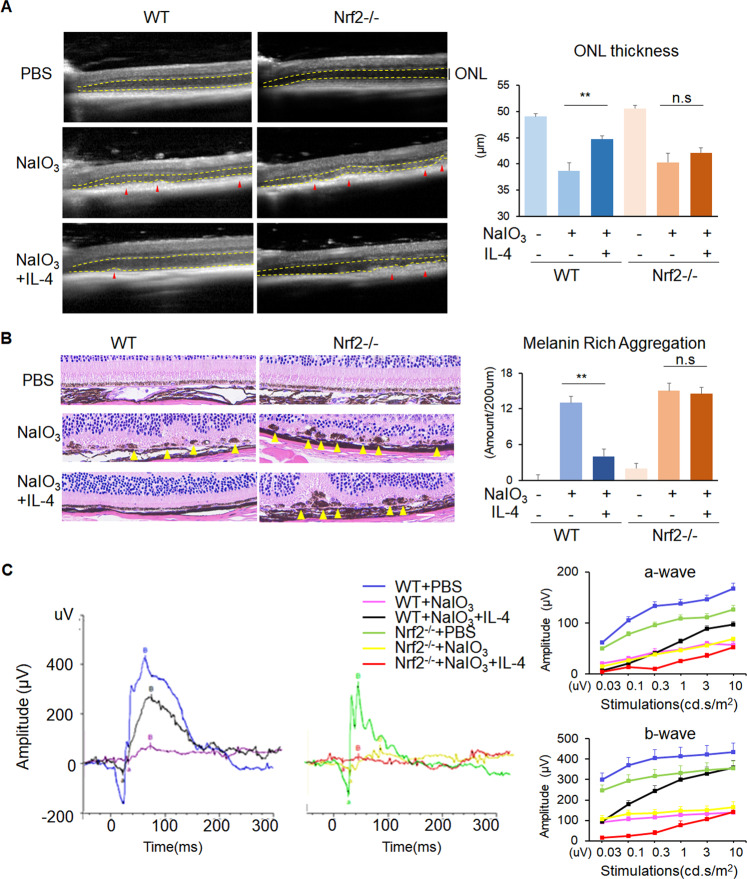
Nrf2 deficiency diminished the protective function of IL-4. **A** In OCT examination, NaIO_3_ model in Nrf2-/-mice presented discontinuity of RPE layer with highly reflective granules (red arrows). Importantly, IL-4 treatment in these mice showed no significant improvement. In addition, the calculated ONL thickness (distance between yellow lines) was reduced in NaIO_3_ model of both WT and Nrf2-/- mice in comparison to those in PBS controls, respectively. IL-4 treatment could partially restore the ONL thickness in NaIO_3_ model of WT mice but not Nrf2-/- mice. *n* = 6. **B** H&E staining revealed melanin rich aggregation (yellow arrows) in the NaIO_3_-RPE from both WT and Nrf2-/- mice. IL-4 treatment rescued RPE injuries in WT-NaIO_3_-mice with much less melanin rich aggregation. However, the IL-4-treated Nrf2-/-NaIO_3_ mice still presented with crumby structure with melanin rich aggregation. Scale bar: 20 μm. *n* = 3. **C** ERG showed increased amplitudes of a- and b- waves after IL-4 treatment in WT-NaIO_3_ retina but not Nrf2-/- NaIO_3_ mice. The Nrf2-/- NaIO_3_ mice with or without IL-4 treatment presented almost extinct waves. *n* = 6. Data are shown as mean ± SEM. **P < 0.01, n.s: no significance, one-way ANOVA test.

To further validate the involvement of Nrf2, we performed the NaIO_3_-induced retinal degeneration in the Nrf2 knockout (Nrf2-/-) mice. The 8 to 12-week-old Nrf2^-/-^-mice did not show signs of degeneration as previously reported [33], and after NaIO_3_ induction, they displayed similar hyperreflective foci (red arrows) in the RPE layer as the WT-NaIO_3_, indicating the Nrf2 depletion alone at the young age would not worsen the pathological process of NaIO_3_-induced retinal degeneration examined by OCT (Fig. 5A). On the other hand, while treatment of IL-4 resulted in rescue of integrity of RPE layer and elevated thickness of ONL in IL-4-WT-NaIO_3_-mice, no recovery of hyperreflective foci and ONL thickness in IL-4-Nrf2^-/-^-NaIO_3_-mice were observed, indicating Nrf2 deficiency abolished the protective effects of IL-4 (Fig. 5A). Although OCT has provided exquisite methods to identify and evaluate multiple layers of ocular structures, the OCT signals could be easily affected by the image quality and structural reflection, leading to biased results. Therefore, we further performed histological analysis by H&E staining, the images revealed clearly that the melanin rich aggregation in the RPE in both WT and Nrf2^-/-^-NaIO_3_-mice with slightly more severe pathology in the Nrf2^-/-^ mice (Fig. 5B). Consistent with the results from the OCT examination, while IL-4 treatment alleviated the RPE injuries in WT- NaIO_3_-mice, it failed to do so in the IL-4-Nrf2^-/-^-NaIO_3_-mice (Fig. 5B). To investigate the functional impact, ERG was also utilized in all individual groups. The WT-NaIO_3_ and Nrf2^-/-^-NaIO_3_- mice presented nearly similar extinct a- and b- waves. Once again, while IL-4-treated WT-NaIO_3_- mice had active waves with elevated amplitudes of a-wave and b-wave, the Nrf2^-/-^-NaIO_3_- mice treated with IL-4 displayed the least amplitudes of a-wave and b-wave (Fig. 5C), indicating IL-4 could not rescue the dysfunction of RPE and photoreceptors without Nrf2. Thus, these data suggested that IL-4 exerted neuroprotective role in a Nrf2-dependent manner.

### IL-4 Induced Anti-oxidative Stress and Anti-inflammatory Genes Expression in Degenerative RPE

**Fig. 6:**
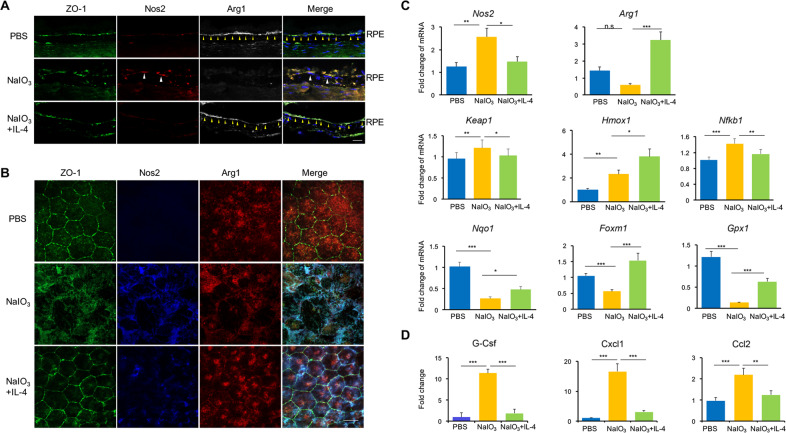
IL-4 induced antioxidant and anti-inflammatory genes expression in vivo. **A** Immunoflorence of cryosection showed high expression of Nos2 in response to NaIO_3_ in the ZO-1+ RPE monolayer, whereas IL-4 treatment decreased Nos2 and increased Arg1 expression. Scale bar: 20 μm. **B** In the RPE flat-mounts, NaIO_3_ disrupted the well-organized hexagonal-shaped RPE cell and induced Nos2 expression elevation, whereas IL-4 treatment reversed it with more organized barrier structures of RPE cells. IL-4 could also suppress the Nos2 expression and promote Arg1 expression within RPE cells. Scale bar: 20 μm. **C** qPCR data showed the reduction of *Nos2* and increase of *Arg1* in the NaIO_3_ + IL-4 group. In addition, IL-4 treatment induced the expressions of anti-oxidative stress-related genes, including *Hmox1*, *Nqo1*, *Foxm1*, and *Gpx1* in the NaIO_3_-retina-RPE complex. *n* = 3. **D** Luminex results showed elevated expression of pro-inflammatory cytokines in the RPE complex of NaIO3-induced model, including G-Csf, Cxcl1, and Ccl2, which decreased after IL-4 treatment. *n* = 3. Data are shown as mean ± SEM. *P < 0.05, **P < 0.01, ***P < 0.001, n.s: no significance, one-way ANOVA test.

IL-4 has been reported to induce the expression of Arg1, which has also been shown to mediate anti-oxidative stress or anti-inflammatory effects [34]. The Nos2 and Arg1 catalysis usually compete against each other to activate a common metabolic pathway and exert the divergent effects [35]. Thus, we examined the expressions of both Nos2 and Arg1 in vivo upon the IL-4 treatment. As shown in the retinal cryosection images of Fig. 6A, whereas NaIO_3_ promoted the expression of Nos2 and down-regulated the Arg1 expression in RPE layer in comparison to PBS control, IL-4 treatment reversed the expression patterns of both genes. The impacts of IL-4 on the expression of Nos2 and Arg1 was further verified via the immunostaining of the RPE flat-mount sheets showing that IL-4 treatment suppressed the expression of Nos2, as well as promoted the Arg1 staining in RPE (Fig. 6B). Consistently, the qPCR analysis also revealed the increased *Arg1* and decreased *Nos2* at the mRNA level (Fig. 6C). Western blot analysis showed that IL-4 treatment also increased Arg1 protein expression while decreased Nos2 expression in the degenerative rd10 retina (Fig. S4A, B). In addition, the expression of a panel of anti-oxidative stress-related genes, including *Hmox1*, *Nqo1*, *Foxm1*, and *Gpx1* were highly increased after IL-4 treatment (Fig. 6C). The luminex assay found the protein expressions of inflammatory genes such as G-Csf, Cxcl1, and Ccl2 were reduced in the IL-4 group (Fig. 6D). These data suggested that IL-4 may regulate the inflammatory and oxidative signaling.

## Discussion

Neurodegenerative diseases are characterized by chronic degenerative pathological changes due to oxidative stress and inflammation [3, 22]. These diseases have very limited therapeutic options, and it is highly needed to explore more effective treatment [36]. In this study, IL-4, a pleiotropic cytokine in immune response, was reported to preserve the structure and neuroretinal function in the NaIO_3_-induced retinal degeneration mouse model and the rd10 mouse, underlining the function and therapeutic potential of IL-4 beyond its classical regulation of Th2-mediated immune responses. RNA-sequencing analysis showed that IL-4 treatment modulated the oxidative stress-related pathways and attenuated the inflammatory injuries. This notion was further confirmed by revealing the requirement of Nrf2 in IL-4-mediated protection using knockout mice. These observations suggested that IL-4 may serve as a therapeutic candidate to treat retinal degeneration via activating Nrf2 signaling.

IL-4, a signature cytokine of Th2-immune response, plays a critical role in the regulation of immune responses, as well as a nonimmune function [37]. In the context of the retina, which consists of multiple well-arranged layers with neurons, glial cells, and RPE cells, the functions of IL-4 are quite complex. IL-4 has been shown to enhance the recruitment of CCR2^+^ bone marrow cells into retinochoroid lesion and promote the choroid neovascularization [38], whereas others reported that IL-4 drove macrophage sFlt-1 production, and the sFlt-1^+^Arg1^+^ macrophages attenuated laser-induced choroidal neovascularization [39]. In this study, we found IL-4 induced IL-4Rα activation and protected the RPE from either inherited or chemically induced degeneration through Nrf2 signaling, expanding the functions of IL-4. Thus, at the context of retinal degeneration, IL-4 exhibited a new neuroprotective feature.

It is noteworthy of the distribution pattern of IL-4 in normal retina indicating that IL-4 is likely derived from microglia based on the shape of IL-4-positive cells with filopodia-like protrusions, closely resembling those of branchy M2 phenotypes [27, 28]. This is also consistent with the human scRNA-seq data (Fig. S1) and previous studies reporting that lymphocytes and myeloid cell were the main cellular sources of IL-4 [40–42]. Interestingly, the expression of IL-4 was abolished by the NaIO_3_ stimulation, suggesting that NaIO_3_ targets on not only RPE cells as previously considered, but also other types as well. In this study, even though we focused on the effects of IL-4 on RPE in vivo and in vitro, the impacts of other types of cells, particularly the microglia, which also expressed IL-4R, would probably respond to this immune cytokine as well [20, 43].

Oxidative stress and inflammation form a vicious circle to promote the neurodegeneration pathogenesis [44]. RPE cells, under oxidative stress initiated by various factor such as aging, play a critical role in the chronic inflammation network leading to the degenerative retina [45]. Our RNA-seq data revealed that IL-4 treatment could not only suppress the expressions of some pro-inflammatory enriched gene sets, but also inhibit the genes of oxidative stress pathways. Specifically, RPE cells were shown to highly express Nos2, a gene to promote reactive nitrogen specie production and mediate inflammation in tissues. In contrast, Arg1, a known competing enzyme, which induces expressions of anti-oxidative and anti-inflammatory genes [46, 47], displayed increased expression upon the IL-4 treatment. The strong inhibitory effects of IL-4 on inflammation and oxidative stress in NaIO_3_-induced RPE might be the underlying mechanism responsible for the potent protection. Consistently, we found Nrf2, a master regulator of cellular defense against oxidative stress [48], was required for IL-4-induced anti-inflammation and anti-oxidative effects. Although Nrf2 has been linked to neuroprotection for long time [49], it still lacks of available pharmacological modulators due to the elusive mechanism of current compounds for inhibiting Nrf2 [50, 51]. Our findings suggest IL-4 might be a new modulator for Nrf2 to treat neurodegenerative diseases.

In conclusion, our study showed that IL-4 was decreased in retinal degeneration, and the application of exogenous IL-4 protected the RPE cells and improved neurofunction through anti-oxidative and anti-inflammatory pathways. The protection of IL-4 was mediated via the IL-4/IL-4Rα-Nrf2 axis. These findings make IL-4 a new potential therapeutic approach for neurodegenerative diseases.

## Materials and methods

### Animals

All the experiments were carried out in accordance with the Association for Research in Vision and Ophthalmology Statement for Use of Animals in Ophthalmic and Vision Research and with the approved guidelines of Animal Care and Use Committee of Zhongshan Ophthalmic Center (Ethics ID: 2018-105). Nrf2-/- mice were bought from the Jackson Laboratory (Strain #:017009), and the mice with rd8 mutation in the Crb1 gene detected by PCR were excluded for this study. Eight- to 12-week-old C57BL/6J and Nrf2-/- mice were used in this study. All mice were age and sex-matched and then randomized into the different groups. Retinal fundus was examined before NaIO_3_ model establishment. To establish the retinal degeneration model, the mice were injected intravenously with 20 mg/kg of NaIO_3_ (Sigma-Aldrich Corp., St. Louis, MO, USA), which was dissolved in phosphate-buffered saline (PBS). The mice receiving the same volume of PBS worked as controls. When receiving treatment, the NaIO_3_ mice were injected intravitreally either with IL-4 (2 μg/μl in 1 μl) or 0.2% BSA as control. Seven days later, the mice were sacrificed by carbon dioxide euthanasia and the eyes were collected for further investigation. The rd10 mouse model, homozygous for the Pde6b^rd10^ on a C57BL/6J background (Strain #:004297) were bought from the Jackson Laboratory, and IL-4 (2 μg/μl in 1 μl) were injected intravitreally every two days for 3 times beginning at P16 for rd10 mice. The same-age C57BL/6J mice received PBS treatment as comparison and they are sacrificed at P25 with eyes collected for further investigations.

### Electroretinogram (ERG) recordings

After dark adaptation overnight, the mice were anesthetized and the pupils were dilated with 0.5% tropicamide. ERG was recorded with gold plated wire loop electrodes contacting the corneal surface as the active electrode. Stainless steel needle electrodes were inserted into the tail serving as ground leads. The mice were exposed to full-field scotopic flashes with intensity of 0.03, 0.1, 0.3,1.0, 3.0, and 10.0 log cd^.^s/m^2^ [52–54]. Amplitudes of a- and b-wave were measured (Celeris-Diagnosys system) as in previous reports [55].

### Visual acuity

The visual acuity of mice was examined as previously described [56]. In brief, the test began when the mouse on top of the platform no longer actively moving around, with contrast set at 100% and a grating of low spatial frequency (0.042 cycles per degree), and the tracking behavior of the mice was observed and recorded. Then the same stimuli were rotated in the anticlockwise direction (thus effectively testing the right eye). The acuity threshold was determined by a staircase method. When the mouse ceased to respond to a particular spatial frequency, a lower frequency grating was presented; when the mouse responded to a frequency, the frequency was increased, until reaching the closest none-responding spatial frequency. The acuity threshold was set as the highest spatial frequency to which the animal responded.

### Optical coherence tomography (OCT)

The images were obtained with SPECTRALIS-OCT (Heidelberg, Germany) with a mouse objective lens. After focusing on the retina with the optic cup placed in the scanning center, OCT scans were acquired in automatic real-time (ART) mode to improve the signal to noise ratio. The quality index of each scan was verified to be more than 28 to show the retinal structures clearly. Twelve scanning-images were recorded in 7.8 × 7.8 mm scanning frame for each eye at 30° field-of-view in a circular order. The images were acquired with 60–100 frames for each scan. To measure the thickness of outer nuclear layer (ONL), quantitative analysis was performed by the Image J software (US National Institutes of Health) as previously described [57]. In brief, the ONL was defined as the distance between the outer plexiform layer to the outer limiting membrane, and was assessed by the averaged thicknesses from 10 locations on each image, measured at 700 μm intervals by 5 locations on each side of the optic disc. The measurements of ONL thickness were performed by experienced investigators in a masked fashion [58–60].

### Retinal fundus imaging and grading

After anesthesia and pupil dilation, the retinal fundus was captured with the Micron IV fundus camera with mouse objective and 50-degree field of view (1.8-mm diameter) centering on the optic cup (Phoenix Research Labs, Pleasanton, CA, USA). Retinal infiltrates and folds were counted in the resulting digital images. The grading system were based on the retinal structural damage scores from 0 to 4. Score 1 refers to 1–4 dot-like lesion or 1 linear lesion, score 2 refers to 5–10 dot-like lesions or 2–3 linear lesions, score 3 refers to >10 dot-like lesions or >3 linear lesions, score 4 refers to confluent linear lesions [23, 61].

### Hematoxylin & Eosin staining and thickness analysis

Eyes were fixed with 4% formalin overnight, embedded in paraffin and cut into 3 μm vertical slices. Sections were washed and treated with hematoxylin buffer followed by eosin solution as previously described [62]. The retinal morphology was observed and evaluated under a microscope (Leica DM4000, Germany). The ONL thickness were measured and the number of melanin rich aggregation in RPE layer was counted in a masked fashion manually and analyzed in 6 fields/eye from 3 eyes. Images were processed and analyzed with Image J software.

### RPE-sclera Isolation, RNA extraction, library construction, and sequencing

Briefly, the anterior segment of the eyeball was cutoff with the lens, vitreous, and the neuroretina carefully removed. The RPE-sclera were collected and put into the Trizol (Thermo Fisher Scientific) for RNA extraction. Total RNA was purified, and the RNA integrity was assessed using the RNA Nano 6000 Assay Kit of the Bioanalyzer 2100 system (Agilent Technologies, CA, USA). Libraries were constructed using the NEBNext® UltraTM RNA Library Prep Kit for Illumina® (NEB, USA) following manufacturer’s recommendations and index codes were added to attribute sequences to each sample. After PCR products were purified (AMPure XP system) and library quality was assessed on the Agilent Bioanalyzer 2100 system, libraries were pooled and sequenced on a cBot Cluster Generation System using TruSeq PE Cluster Kit v3-cBot-HS (Illumia).

### RNA-sequencing data processing

Raw data (raw reads) of fastq format were firstly processed through in-house perl scripts with clean data (clean reads) obtained and low-quality reads removed. Retained reads were then aligned to mus musculus genome using STAR (v 2.7.3a) in the pairend mode with default parameters. Reads number mapped to each gene was counted using Hisat2 v2.0.5. The output matrix from Hisat2 was input into the DESeq2 (v1.26.0) for identification of differentially expressed genes between diabetes group and control group. We determined differentially expressed genes with the criteria of absolute fold change >2 and false discovery rate (FDR) adjusted *P*-value < 0.05. In addition, log-fold change of all genes between two groups were used to conduct Gene Ontology (GO) enrichment analysis. GO terms with corrected *P*-value less than 0.05 were considered significantly enriched by differential expressed genes. GO and circos plots were generated by ggplot2 (v 3.2.1) and clusterProfiler. Gene-set enrichment analysis (GSEA) was conducted using the GSEA software (http://www.gsea-msigdb.org/gsea/index.jsp). The NaIO_3_ induced RPE was class A, and IL-4 treated NaIO_3_ RPE was class B. Gene-set permutations were performed 1000 times for each analysis. The cutoff values were predefined as FDR < 0.25. Our sequencing data are available at Gene Expression Omnibus under the accession number GSE193808.

### Analysis of scRNA-seq data

The scRNA-seq datasets were retrieved from NCBI’s Gene Expression Omnibus (https://www.ncbi.nlm.nih.gov/geo/query/acc.cgi?acc= GSE135922, No. GSE135922), which included sequenced single cells from human RPE/choroid of both healthy controls and AMD patients (Donors 1–3) as previously described [25, 26]. After quantification and normalization for each sample, unsupervised clustering analysis was further performed to identify the highly variable genes. Then principal component analysis was performed on the highly variable genes. Non-linear dimensional reduction (tSNE) was used to visualize clustering results based on the top PCs. Graph-based clustering was performed to cluster cells by using the FindNeighbors and FindClusters function in Seurat (clustering resolution = 1.0). We screened the top-ranking marker gene lists for each cluster to perform cluster annotation to specific cell types. Transcriptomic differences of IL-4 between different cell types were statistically compared and analyzed using visualization tools of DotPlot. The expression level of IL-4 between normal and AMD patient was visualized as VlnPlot.

### ARPE-19 cell line culture

The human ARPE-19 cell line was purchased from ScienCell, China. The RPE cells were treated with IL-4 (100 ng/ml, dissolved in PBS, 96-214-14-50, PeproTech) or PBS for 2 h and then stimulated with hydrogenperoxide (H_2_O_2_, 300 μM). Twenty-four hours later, the cells were harvested for further analysis.

### Quantitative PCR analysis and western blotting

The qPCR was performed as previously described [63]. The total volume of 20 μl contained 2 μl of cDNA, 10 μl of 2×SYBR Premix Ex Taq, 7 μl ddH_2_O and 10 μmol/l of the primer pairs. The sequences of the used primers were listed in the supplement Table 1 and qPCR was performed using Roche LightCycler480 II. The western blotting was performed as previously described [64]. Primary antibodies included anti-IL-4 antibody (Santa Cruz, sc-53084), anti-IL-4Rα antibody (Santa Cruz, sc-28361), anti-Nrf2 (Abcam, ab137550), anti-p-Nrf2 antibody (Abcam, ab76026), anti-Nos2 (Santa Cruz, sc-7271), anti-Arg1 (Santa Cruz, sc-18355), and anti-β-actin (Cell Signaling Technology, 4970S).

### Immunofluorescence

For cryosections, eyes were removed carefully and embedded in optimal cutting temperature compound (Sakura Fine Technical). The frozen samples were then sliced transversely (8 μm) with a cryostat (Leica CM1950) at –20 °C. Only the cross-sections throughout optic nerve were used for staining and analysis. For retinal-RPE flat-mount, the eyes were fixed in 4% PFA for 60 min at room temperature and the RPE-sclera complex were dissected out. The cryosection, RPE flat-mount sheet samples and ARPE cells were blocked with 0.5% Triton-X100/5% BSA and incubated with primary antibodies overnight at 4 °C. The primary antibodies included anti-ZO-1 antibody (Santa Cruz, sc-33725), anti-Arg1 antibody (Santa Cruz, sc-18355), anti-Nos2 antibody (Santa Cruz, sc-7271), anti-IL-4Ra antibody (Santa Cruz, sc-28361), anti-IL-4 antibody (Santa Cruz, sc-53084), and anti-p-Nrf2 antibody (Abcam, ab76026). Then they were incubated with secondary antibodies for 1 h and counterstained with DAPI (Invitrogen, D1306) for 5 min before mounted. Negative control was done by omission of the primary antibody. The images were obtained using a confocal microscope (LSM880, Carl Zeiss).

### Multiplex immunoassay

Retinal-RPE-mixture protein were collected and the concentration were measured by BCA protein assay and diluted to the concentration of 1 mg/ml. Cytokine/chemokine levels were analyzed using MilliPlex map Mouse Angiogenesis/Growth Factor Magnetic Bead Panel (15 plex) from Millipore according to manufacturer’s instructions [65]. All data was collected on the Luminex 200 at Laizee Biotech CO., LTD (Beijing, China).

### Statistics

All experiments including immunostaining, qPCR, and WB were repeated at least three times with representative results shown in the figures. Data were presented as mean ± standard error of mean (SEM) with the number of samples provided in the figure legends, and were analyzed statistically using an unpaired Student’s *t*-test (two groups) or one-way ANOVA (>2 groups) with a Turkey’s post hoc comparisons test. *P*-values < 0.05 were considered statistically significant.

## Supplementary information

Supplementary material

Original Data File

Checklist

## Data Availability

RNA-Sequencing data have been deposited in the Gene Expression Omnibus database with accession numbers: GSE193808. The human ScRNA-seq data were downloaded from the NCBI Gene Expression Omnibus database (GSE135922) published previously [25, 26]. Additional data generated or analyzed during this study are included in this published article.

## References

[1] Takeda A, Baffi JZ, Kleinman ME, Cho WG, Nozaki M, Yamada K (2009). CCR3 is a target for age-related macular degeneration diagnosis and therapy. Nature..

[2] Kaarniranta K, Uusitalo H, Blasiak J, Felszeghy S, Kannan R, Kauppinen A (2020). Mechanisms of mitochondrial dysfunction and their impact on age-related macular degeneration. Prog Retinal Eye Res.

[3] Ferrington DA, Sinha D, Kaarniranta K (2016). Defects in retinal pigment epithelial cell proteolysis and the pathology associated with age-related macular degeneration. Prog Retinal Eye Res.

[4] Saini JS, Corneo B, Miller JD, Kiehl TR, Wang Q, Boles NC (2017). Nicotinamide ameliorates disease phenotypes in a human iPSC model of age-related macular degeneration. Cell Stem Cell.

[5] da Cruz L, Fynes K, Georgiadis O, Kerby J, Luo YH, Ahmado A (2018). Phase 1 clinical study of an embryonic stem cell-derived retinal pigment epithelium patch in age-related macular degeneration. Nat Biotechnol.

[6] Mettu PS, Allingham MJ, Cousins SW (2021). Incomplete response to Anti-VEGF therapy in neovascular AMD: Exploring disease mechanisms and therapeutic opportunities. Prog Retinal Eye Res.

[7] Jansen JH, Fibbe WE, Willemze R, Kluin-Nelemans JC (1990). Interleukin-4. A regulatory protein. Blut..

[8] Nakayama T, Hirahara K, Onodera A, Endo Y, Hosokawa H, Shinoda K (2017). Th2 cells in health and disease. Annu Rev Immunol.

[9] Gowthaman U, Chen JS, Zhang B, Flynn WF, Lu Y, Song W, et al. Identification of a T follicular helper cell subset that drives anaphylactic IgE. Science. 2019;365:eaaw6433. 10.1126/science.aaw6433 PMC6901029 31371561

[10] Huang SC, Smith AM, Everts B, Colonna M, Pearce EL, Schilling JD (2016). Metabolic reprogramming mediated by the mTORC2-IRF4 signaling axis is essential for macrophage alternative activation. Immunity..

[11] Blauvelt A, de Bruin-Weller M, Gooderham M, Cather JC, Weisman J, Pariser D (2017). Long-term management of moderate-to-severe atopic dermatitis with dupilumab and concomitant topical corticosteroids (LIBERTY AD CHRONOS): a 1-year, randomised, double-blinded, placebo-controlled, phase 3 trial. Lancet.

[12] Nguyen KD, Qiu Y, Cui X, Goh YP, Mwangi J, David T (2011). Alternatively activated macrophages produce catecholamines to sustain adaptive thermogenesis. Nature..

[13] Ji L, Zhao X, Zhang B, Kang L, Song W, Zhao B (2019). Slc6a8-mediated creatine uptake and accumulation reprogram macrophage polarization via regulating cytokine responses. Immunity..

[14] Willenborg S, Sanin DE, Jais A, Ding X, Ulas T, Nüchel J (2021). Mitochondrial metabolism coordinates stage-specific repair processes in macrophages during wound healing. Cell Metab.

[15] Shintani Y, Ito T, Fields L, Shiraishi M, Ichihara Y, Sato N (2017). IL-4 as a repurposed biological drug for myocardial infarction through augmentation of reparative cardiac macrophages: proof-of-concept data in mice. Sci Rep.

[16] Bankaitis KV, Fingleton B (2015). Targeting IL4/IL4R for the treatment of epithelial cancer metastasis. Clin Exp Metastasis.

[17] Hurdayal R, Brombacher F (2017). Interleukin-4 receptor alpha: from innate to adaptive immunity in murine models of cutaneous leishmaniasis. Front Immunol.

[18] Yamani A, Wu D, Waggoner L, Noah T, Koleske AJ, Finkelman F (2018). The vascular endothelial specific IL-4 receptor alpha-ABL1 kinase signaling axis regulates the severity of IgE-mediated anaphylactic reactions. J Allergy Clin Immunol.

[19] Knipper JA, Willenborg S, Brinckmann J, Bloch W, Maaß T, Wagener R (2015). Interleukin-4 receptor α signaling in myeloid cells controls collagen fibril assembly in skin repair. Immunity..

[20] Zhao Z, Liang Y, Liu Y, Xu P, Flamme-Wiese MJ, Sun D (2017). Choroidal γδ T cells in protection against retinal pigment epithelium and retinal injury. Faseb.

[21] Bhutto IA, Ogura S, Baldeosingh R, McLeod DS, Lutty GA, Edwards MM (2018). An acute injury model for the phenotypic characteristics of geographic atrophy. Investig Ophthalmol Vis Sci.

[22] Ambati J, Atkinson JP, Gelfand BD (2013). Immunology of age-related macular degeneration. Nat Rev Immunol.

[23] Moriguchi M, Nakamura S, Inoue Y, Nishinaka A, Nakamura M, Shimazawa M (2018). Irreversible photoreceptors and RPE cells damage by intravenous sodium iodate in mice is related to macrophage accumulation. Investig Ophthalmol Vis Sci.

[24] Bowes Rickman C, Farsiu S, Toth CA, Klingeborn M (2013). Dry age-related macular degeneration: mechanisms, therapeutic targets, and imaging. Investig Ophthalmol Vis Sci.

[25] Voigt AP, Mulfaul K, Mullin NK, Flamme-Wiese MJ, Giacalone JC, Stone EM (2019). Single-cell transcriptomics of the human retinal pigment epithelium and choroid in health and macular degeneration. Proc Natl Acad Sci USA.

[26] Sterling JK, Baumann B, Foshe S, Voigt A, Guttha S, Alnemri A (2022). Inflammatory adipose activates a nutritional immunity pathway leading to retinal dysfunction. Cell Rep.

[27] Zhou T, Huang Z, Sun X, Zhu X, Zhou L, Li M (2017). Microglia polarization with M1/M2 phenotype changes in rd1 mouse model of retinal degeneration. Front Neuroanat.

[28] Hu F, Ma Y, Xu Z, Zhang S, Li J, Sun X (2022). Single-cell RNA-seq reveals the cellular diversity and developmental characteristics of the retinas of an infant and a young child. Front Cell Dev Biol.

[29] Niedzielska E, Smaga I, Gawlik M, Moniczewski A, Stankowicz P, Pera J (2016). Oxidative stress in neurodegenerative diseases. Mol Neurobiol.

[30] Lin MT, Beal MF (2006). Mitochondrial dysfunction and oxidative stress in neurodegenerative diseases. Nature..

[31] Buendia I, Michalska P, Navarro E, Gameiro I, Egea J, Leon R (2016). Nrf2-ARE pathway: An emerging target against oxidative stress and neuroinflammation in neurodegenerative diseases. Pharmacol Ther.

[32] Crunkhorn S (2012). Deal watch: Abbott boosts investment in NRF2 activators for reducing oxidative stress. Nat Rev Drug Discov.

[33] Zhao Z, Chen Y, Wang J, Sternberg P, Freeman ML, Grossniklaus HE (2011). Age-related retinopathy in NRF2-deficient mice. PLoS ONE.

[34] Zhang J, Rong P, Zhang L, He H, Zhou T, Fan Y, et al. IL4-driven microglia modulate stress resilience through BDNF-dependent neurogenesis. Sci Adv. 2021;7:eabb9888. 10.1126/sciadv.abb9888 PMC7968840 33731342

[35] Bronte V, Serafini P, Mazzoni A, Segal DM, Zanovello P (2003). L-arginine metabolism in myeloid cells controls T-lymphocyte functions. Trends Immunol.

[36] Chew EY, Clemons TE, Agrón E, Launer LJ, Grodstein F, Bernstein PS (2015). Effect of omega-3 fatty acids, lutein/zeaxanthin, or other nutrient supplementation on cognitive function: the AREDS2 randomized clinical trial. JAMA..

[37] Wynn TA (2015). Type 2 cytokines: mechanisms and therapeutic strategies. Nat Rev Immunol.

[38] Baba T, Miyazaki D, Inata K, Uotani R, Miyake H, Sasaki SI, et al. Role of IL-4 in bone marrow driven dysregulated angiogenesis and age-related macular degeneration. eLife. 2020;9:e54257. 10.7554/eLife.54257 PMC7200155 32366355

[39] Wu WK, Georgiadis A, Copland DA, Liyanage S, Luhmann UF, Robbie SJ (2015). IL-4 regulates specific Arg-1(+) macrophage sFlt-1-mediated inhibition of angiogenesis. Am J Pathol.

[40] Bonnard B, Ibarrola J, Lima-Posada I, Fernández-Celis A, Durand M, Genty M, et al. Neutrophil gelatinase-associated lipocalin from macrophages plays a critical role in renal fibrosis via the CCL5 (chemokine ligand 5)-Th2 cells-IL4 (Interleukin 4) pathway. Hypertension. 2022;79:352–364. 10.1161/HYPERTENSIONAHA.121.17712 34794340

[41] Shinkai K, Mohrs M, Locksley RM (2002). Helper T cells regulate type-2 innate immunity in vivo. Nature..

[42] Sabin EA, Kopf MA, Pearce EJ (1996). Schistosoma mansoni egg-induced early IL-4 production is dependent upon IL-5 and eosinophils. The. J Exp Med.

[43] Schwarzer P, Kokona D, Ebneter A, Zinkernagel MS (2020). Effect of inhibition of colony-stimulating factor 1 receptor on choroidal neovascularization in mice. Am J Pathol.

[44] Potilinski MC, Tate PS, Lorenc VE, Gallo JE (2021). New insights into oxidative stress and immune mechanisms involved in age-related macular degeneration tackled by novel therapies. Neuropharmacology..

[45] Datta S, Cano M, Ebrahimi K, Wang L, Handa JT (2017). The impact of oxidative stress and inflammation on RPE degeneration in non-neovascular AMD. Prog Retinal Eye Res.

[46] Rath M, Muller I, Kropf P, Closs EI, Munder M (2014). Metabolism via arginase or nitric oxide synthase: two competing arginine pathways in macrophages. Front Immunol.

[47] Vats D, Mukundan L, Odegaard JI, Zhang L, Smith KL, Morel CR (2006). Oxidative metabolism and PGC-1beta attenuate macrophage-mediated inflammation. Cell Metab.

[48] Vomund S, Schäfer A, Parnham MJ, Brüne B, von Knethen A. Nrf2, the Master Regulator of Anti-Oxidative Responses. Int J Mol Sci. 2017;18:2772. 10.3390/ijms18122772 PMC5751370 29261130

[49] Yang Y, Ng TK, Ye C, Yip YW, Law K, Chan SO (2014). Assessing sodium iodate-induced outer retinal changes in rats using confocal scanning laser ophthalmoscopy and optical coherence tomography. Investig Ophthalmol Vis Sci.

[50] Kieseier BC, Wiendl H (2015). Nrf2 and beyond: deciphering the mode of action of fumarates in the inflamed central nervous system. Acta Neuropathol.

[51] Robledinos-Anton N, Fernandez-Gines R, Manda G, Cuadrado A (2019). Activators and inhibitors of NRF2: a review of their potential for clinical development. Oxid Med Cell Longev.

[52] Antes R, Ezra-Elia R, Weinberger D, Solomon A, Ofri R, Michaelson DM (2013). ApoE4 induces synaptic and ERG impairments in the retina of young targeted replacement apoE4 mice. PLoS ONE.

[53] Robson AG, Frishman LJ, Grigg J, Hamilton R, Jeffrey BG, Kondo M (2022). ISCEV Standard for full-field clinical electroretinography (2022 update). Doc Ophthalmol.

[54] Biel M, Seeliger M, Pfeifer A, Kohler K, Gerstner A, Ludwig A (1999). Selective loss of cone function in mice lacking the cyclic nucleotide-gated channel CNG3. Proc Natl Acad Sci USA.

[55] Albrecht NE, Alevy J, Jiang D, Burger CA, Liu BI, Li F (2018). Rapid and integrative discovery of retina regulatory molecules. Cell Rep.

[56] Mahato B, Kaya KD, Fan Y, Sumien N, Shetty RA, Zhang W (2020). Pharmacologic fibroblast reprogramming into photoreceptors restores vision. Nature..

[57] Dysli C, Enzmann V, Sznitman R, Zinkernagel MS (2015). Quantitative analysis of mouse retinal layers using automated segmentation of spectral domain optical coherence tomography images. Transl Vis Sci Technol.

[58] Dromel PC, Singh D, Andres E, Likes M, Kurisawa M, Alexander-Katz A (2021). A bioinspired gelatin-hyaluronic acid-based hybrid interpenetrating network for the enhancement of retinal ganglion cells replacement therapy. NPJ Regenerat Med.

[59] Knott EJ, Sheets KG, Zhou Y, Gordon WC, Bazan NG (2011). Spatial correlation of mouse photoreceptor-RPE thickness between SD-OCT and histology. Exp Eye Res.

[60] Zeng Y, Petralia RS, Vijayasarathy C, Wu Z, Hiriyanna S, Song H (2016). Retinal structure and gene therapy outcome in retinoschisin-deficient mice assessed by spectral-domain optical coherence tomography. Investig Ophthalmol Vis Sci.

[61] Xu H, Koch P, Chen M, Lau A, Reid DM, Forrester JV (2008). A clinical grading system for retinal inflammation in the chronic model of experimental autoimmune uveoretinitis using digital fundus images. Exp Eye Res.

[62] Huang Z, Zhou T, Sun X, Zheng Y, Cheng B, Li M (2018). Necroptosis in microglia contributes to neuroinflammation and retinal degeneration through TLR4 activation. Cell Death Differ.

[63] Zhou T, Liu Y, Yang Z, Ni B, Zhu X, Huang Z (2021). IL-17 signaling induces iNOS+ microglia activation in retinal vascular diseases. Glia.

[64] He C, Liu Y, Huang Z, Yang Z, Zhou T, Liu S, et al. A specific RIP3(+) subpopulation of microglia promotes retinopathy through a hypoxia-triggered necroptotic mechanism. Proc Natl Acad Sci USA. 2021;118:e2023290118. 10.1073/pnas.2023290118 PMC7980367 33836603

[65] Lai P, Chen X, Guo L, Wang Y, Liu X, Liu Y (2018). A potent immunomodulatory role of exosomes derived from mesenchymal stromal cells in preventing cGVHD. J Hematol Oncol.

