# Supplementary material for: IL-4 induces reparative phenotype of RPE cells and protects against retinal neurodegeneration via Nrf2 activation
Source: Cell Death Dis. 2022 Dec 20;13(12):1056. doi: 10.1038/s41419-022-05433-0 (PMC9768119; doi:10.1038/s41419-022-05433-0)
Supplement: Supplementary file 1 — Supplementary material [file 41419_2022_5433_MOESM1_ESM.pdf]

Supplementary Information

IL-4 induces reparative phenotype of RPE cells and

protects against retinal neurodegeneration via Nrf2 activation

Tian Zhou[1,2], Ziqi Yang[1,2], Biyan Ni[1,2], Hong Zhou[1], Huiyi Xu[1], Xiaojing Lin[1], Yingmin Li[1], Chunqiao Liu[1], Rong Ju[1], Jian Ge[1], Chang He[1,3], Xialin Liu[1,3]

[1] State Key Laboratory of Ophthalmology, Zhongshan Ophthalmic Center, Sun Yat-sen University, Guangdong Provincial Key Laboratory of Ophthalmology and Visual Science, Guangzhou 510060, China.

[2] These authors contributed equally to this work.

[3] Correspondence:

Xialin Liu (liuxl28@mail.sysu.edu.cn); Chang He (hech33@mail.sysu.edu.cn)

Running title: IL-4 protects RPE cells via Nrf2

Supplementary Figures 1-4；

Supplementary Table 1；

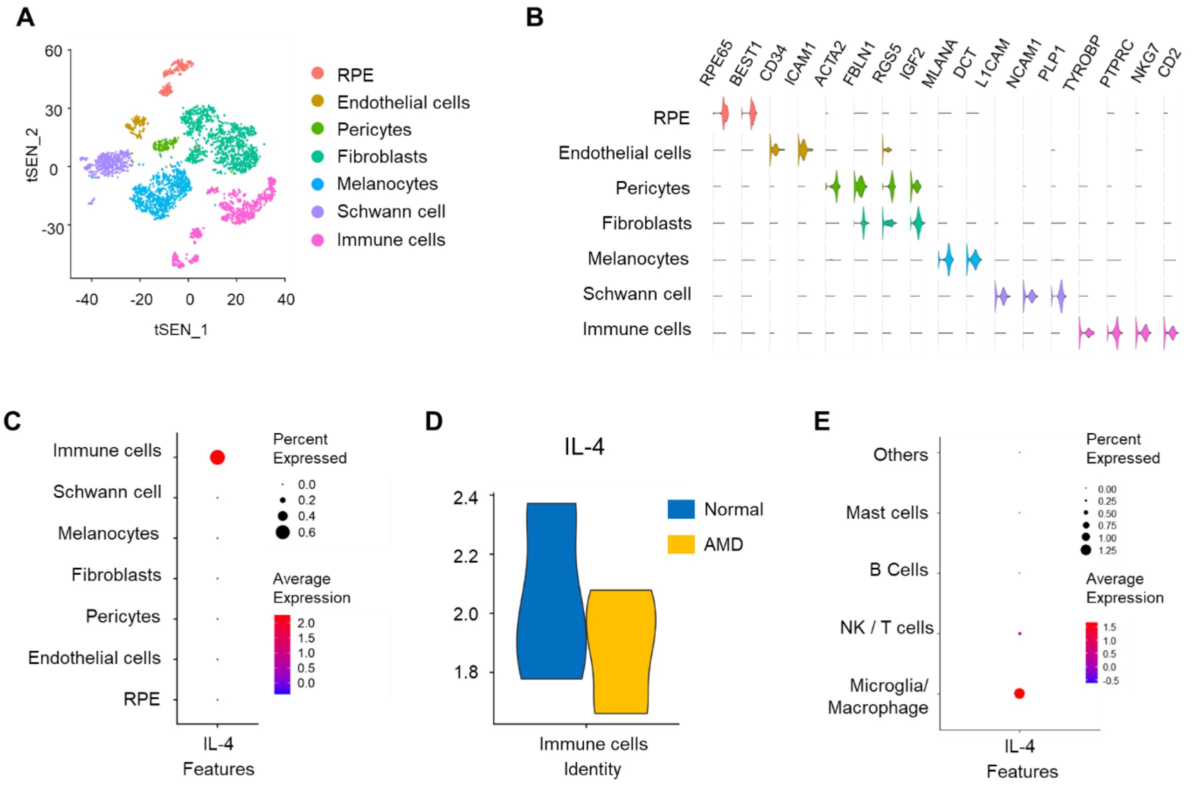

Figure S1. scRNA-seq analysis of human RPE/choroid complex showed decreased IL-4 expression in AMD patients.

(A) t-SNE for scRNA-seq data of human RPE/choroid complex from AMD patient and heathy control. The sub-clustering reveals various cell populations. Each dot represents one cell. (B) The marker genes expression of every cell cluster by scRNA-seq. (C) Dot plot representing expression levels and frequencies of IL-4 among different cell clusters. Among them, IL-4 was specifically expressed in immune cells in comparison to other cell types. (D) Violin Plot showing the decreased IL-4 expression in AMD patient in comparison to normal control. (E) Immune cells were further interrogated into different subclusters based on their markers, including microglia/macrophage, NK/T cells, B cells, mast cells, and others. Dot plot showing that IL-4 was mainly enriched in microglia/macrophage.

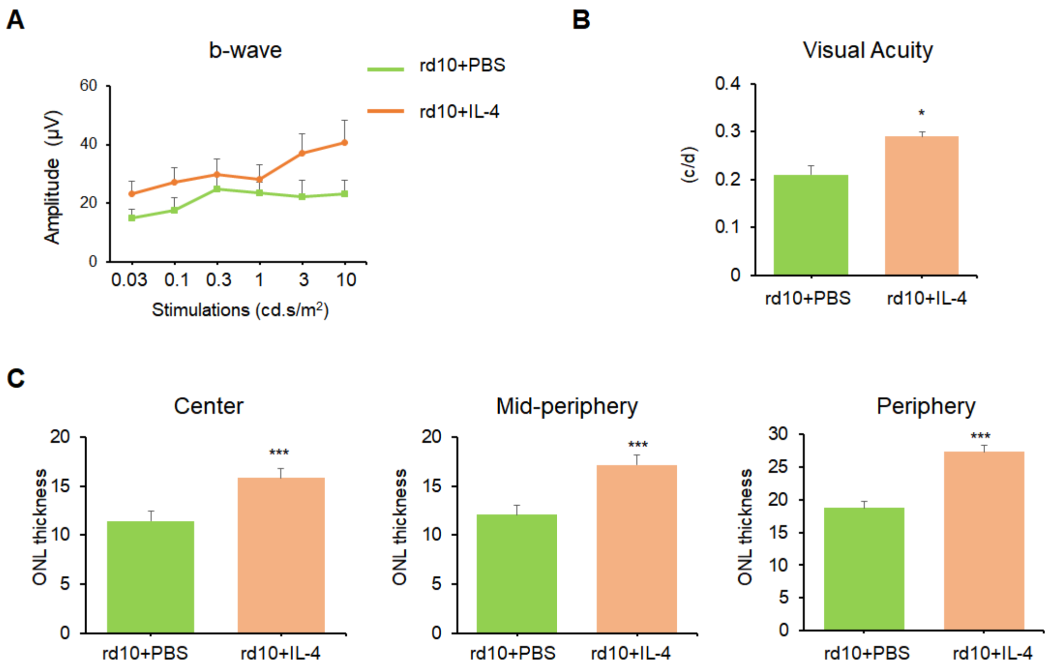

Figure S2. IL-4 protected the retina against neurodegeneration in the rd10 mice.

(**A**) ERG examinations showed IL-4 treatment protected the retinal neurofunction with increased amplitudes of b-waves in rd10 mice. n = 6 (**B**) The measurement of visual acuity demonstrated improved visual acuity response after IL-4 treatment. n = 6. (C) The ONL thickness was calculated from H&E staining at the center, mid-periphery and periphery areas. IL-4 treatment increased the ONL thickness, particularly in the periphery area. n = 3. Data are shown as mean ± SEM, *P < 0.01, **P < 0.05, ***P < 0.001, n.s: not significant, one-way ANOVA test.

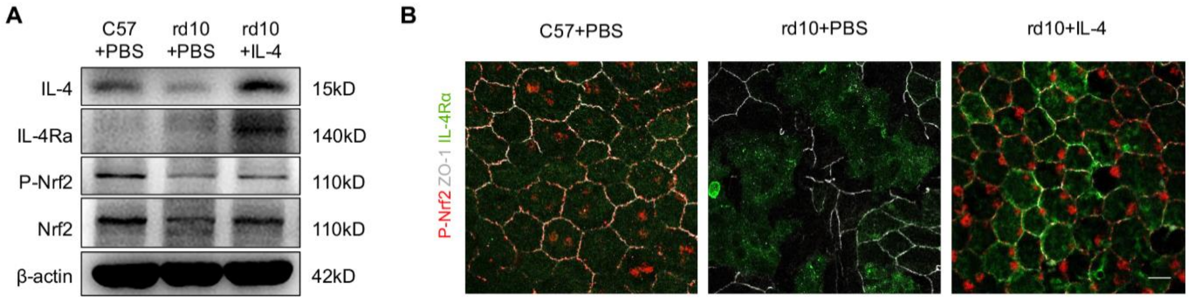

Figure S3. IL-4 upregulated the IL-4R and activated Nrf2 signaling in rd10 mice.

(A) Western blotting analysis showed that IL-4Ra and p-Nrf2 expression were increased in RPE complex from the IL-4 treated rd10 mice. (B) Representative images of RPE flat-mount sheet revealed that IL-4 treatment up-regulated the expression of IL-4Ra alongside the RPE tight junction marker ZO-1. The p-Nrf2 was also accumulated alongside the hexagonal-shaped RPE in the IL-4 treated rd10 mice. Scale bar: 20 µm.

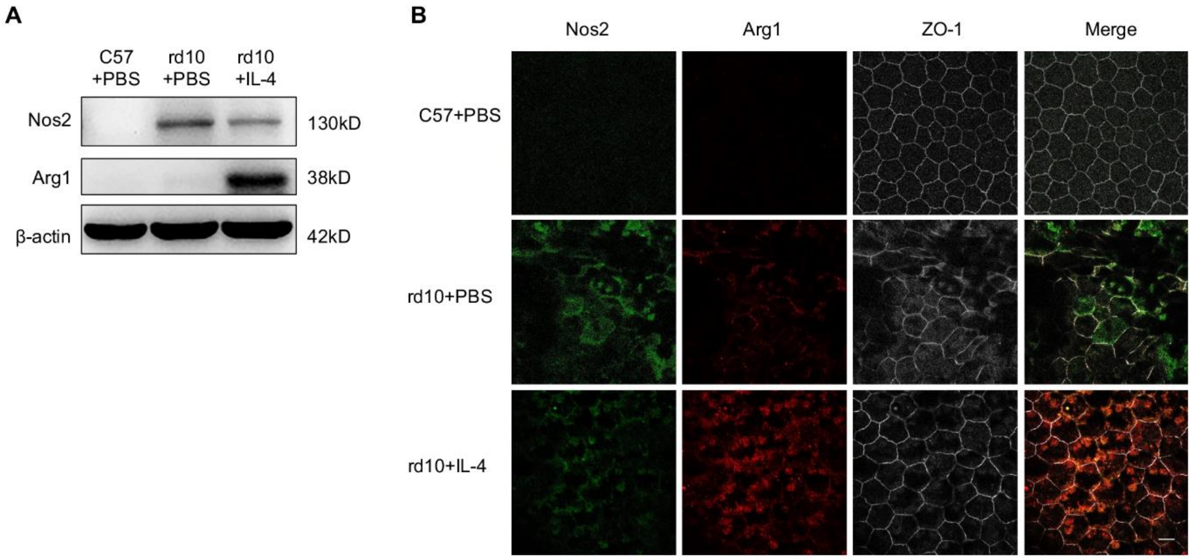

Figure S4. IL-4 suppressed the Nos2 and promoted Arg1 expression in rd10 mice.

(A) Western blotting analysis of RPE complex showed high expression of Nos2 in rd10 mice, whereas IL-4 treatment suppressed the expression of Nos2 and promoted the expression of Arg1. (B) In the RPE flat-mounts, rd10 mice also displayed elevated Nos2 expression alongside the disrupted hexagonal-shaped RPE cell. After IL-4 treatment, the barrier structures of RPE cells were preserved with decreased Nos2 expression and increased Arg1 level. Scale bar: 20 μm.

Supplement Table 1. Sequences of the primers used in this study.

Gene name	Orientation	Primer sequence (5' to 3')	Species
Nos2	Forward	TTCAGTATCACAACCTCAGCAAG	Mouse
	Reverse	TGGACCTGCAAGTTAAAATCCC	Mouse
Il-6	Forward	TTCAGGCAGGCAGTATCACTC	Mouse
	Reverse	GAAGGTCCACGGGAAAGACAC	Mouse
Arg1	Forward	TCAGCGTGTCCAAACACTGAG	Mouse
	Reverse	CGCCAAGGGAGTTAAAGACTT	Mouse
Il-4ra	Forward	TCTGCATCCCGTTGTTTTGC	Mouse
	Reverse	GCACCTGTGCATCCTGAATG	Mouse
Il-4	Forward	GGTCTCAACCCCCAGCTAGT	Mouse
	Reverse	GCCGATGATCTCTCTCAAGTGAT	Mouse
Il-10	Forward	TCCAGCCTTACATCCACCTC	Mouse
	Reverse	GCTGCTGTCTGTGGATTTCA	Mouse
Tnf-α	Forward	CCCTCACACTCAGATCATCTTCT	Mouse
	Reverse	GCTACGACGTGGGCTACAG	Mouse
Ifn-γ	Forward	ATGAACGCTACACACTGCATC	Mouse
	Reverse	CCATCCTTTTGCCAGTTCCTC	Mouse
Cxcl1	Forward	CTGGGATTCACCTCAAGAACATC	Mouse
	Reverse	CAGGGTCAAGGCAAGCCTC	Mouse
Cxcl2	Forward	CCAACCACCAGGCTACAGG	Mouse
	Reverse	GCGTCACACTCAAGCTCTG	Mouse

Gene name	Orientation	Primer sequence (5' to 3')	Species
Cxcl10	Forward	CCAAGTGCTGCCGTCATTTTC	Mouse
	Reverse	GGCTCGCAGGGATGATTTCAA	Mouse
Cxcr1	Forward	TCTGGACTAATCCTGAGGGTG	Mouse
	Reverse	GCCTGTTGGTTATTGGAACTCTC	Mouse
Cxcr2	Forward	ATGCCCTCTATTCTGCCAGAT	Mouse
	Reverse	GTGCTCCGGTTGTATAAGATGAC	Mouse
Hmox1	Forward	AAGCCGAGAATGCTGAGTTCA	Mouse
	Reverse	GCCGTGTAGATATGGTACAAGGA	Mouse
Keap1	Forward	TGCCCCTGTGGTCAAAGTG	Mouse
	Reverse	GGTTCGGTTACCGTCCTGC	Mouse
Nfkb1	Forward	GGAGGCATGTTCGGTAGTGG	Mouse
	Reverse	CCCTGCGTTGGATTTCGTG	Mouse
Nqo1	Forward	AGGATGGGAGGTACTCGAATC	Mouse
	Reverse	AGGCGTCCTTCCTTATATGCTA	Mouse
Fomx1	Forward	CTGATTCTCAAAAGACGGAGGC	Mouse
	Reverse	TTGATAATCTTGATTCCGGCTGG	Mouse
Gpx1	Forward	AGTCCACCGTGTATGCCTTCT	Mouse
	Reverse	GAGACGCGACATTCTCAATGA	Mouse
Gapdh	Forward	GCCAAGGCTGTGGGCAAGGT	Mouse
	Reverse	TCTCCAGGCGGCACGTCAGA	Mouse
