# Supplementary figures and images for: IL-4 induces reparative phenotype of RPE cells and protects against retinal neurodegeneration via Nrf2 activation
Source: Cell Death Dis. 2022 Dec 20;13(12):1056. doi: 10.1038/s41419-022-05433-0 (PMC9768119; doi:10.1038/s41419-022-05433-0)

Figure 1D

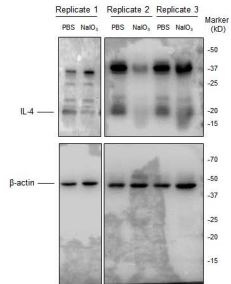

Figure 4B

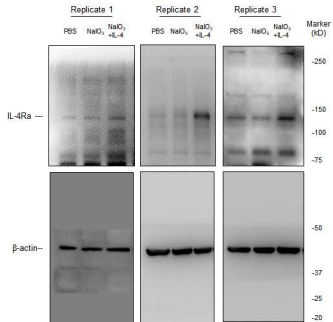

Figure 4D

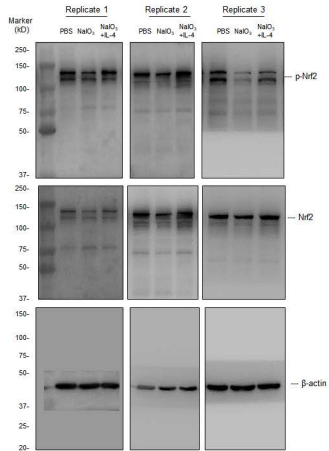

Figure S3A, S4A

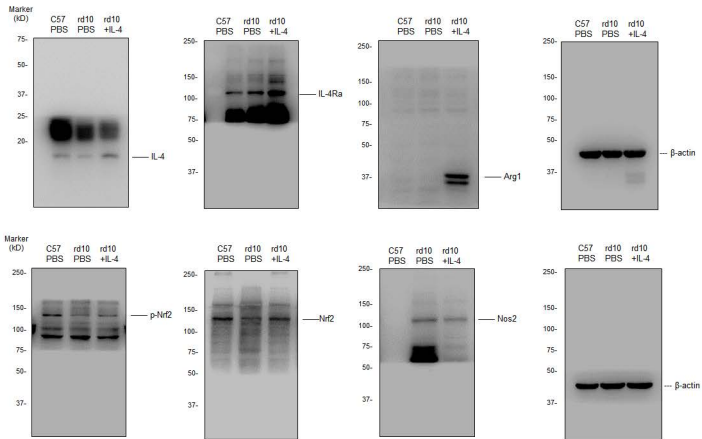

Supplement: Supplementary file 2 — Original Data File [file 41419_2022_5433_MOESM2_ESM.pdf]
